# “I don’t really know how to help her.” Family caregivers’ capabilities, opportunities and motivations to provide hearing support to long-term care home residents with dementia

**DOI:** 10.1080/09638288.2024.2384630

**Published:** 2024-08-07

**Authors:** Hannah Cross, Christopher J. Armitage, Angela Clayton-Turner, Sandra Barker, Piers Dawes, Iracema Leroi, Rebecca E. Millman

**Affiliations:** a Manchester Centre for Audiology and Deafness, School of Health Sciences, University of Manchester, Manchester, United Kingdom; b NIHR Manchester Biomedical Research Centre, Manchester University Hospitals NHS Foundation Trust, Manchester Academic Health Science Centre, Manchester, United Kingdom; c Manchester Centre for Health Psychology, University of Manchester, Manchester, United Kingdom; d Manchester University NHS Foundation Trust, Manchester Academic Health Science Centre, Manchester, United Kingdom; e NIHR Greater Manchester Patient Safety Research Collaboration, University of Manchester, Manchester, United Kingdom; f Alzheimer’s Society, London, United Kingdom; g Centre for Hearing Research (CHEAR), School of Health and Rehabilitation Sciences, University of Queensland, Brisbane, Australia; h Global Brain Health Institute and School of Medicine, Trinity College Dublin, Dublin, Ireland

**Keywords:** Hearing loss, hearing aids, audiology, dementia, care home, behaviour change wheel

## Abstract

**Purpose:**

Hearing loss is highly prevalent in long-term care home (LTCH) residents with dementia (“residents”) and exacerbates confusion and communication difficulties. Residents rely on caregivers, including family, for hearing-related care. This study aims to understand the drivers of family caregivers’ provision of hearing support to LTCH residents using the Behaviour Change Wheel.

**Materials and Methods:**

This exploratory two-stage study was guided by the Capability, Opportunity, Motivation-Behaviour (COM-B) model and the Theoretical Domains Framework (TDF). A self-report survey (*N* = 87) and interviews (*N* = 6) explored drivers behind the provision of hearing support. Quantitative data were analysed using descriptive statistics and a within-participants ANOVA. Deductive coding of TDF domains alongside thematic analysis was used for qualitative data.

**Results:**

Provision of hearing support was variable. Gaps in family caregivers’ psychological capability, reflective motivation and physical opportunity were identified. Barriers included lacking knowledge, unclear caregiver responsibilities, deprioritising hearing support, COVID-19 restrictions and fragmented collaborations with audiology services.

**Conclusions:**

Future behaviour-change interventions to facilitate family caregivers’ provision of hearing support to LTCH residents should include: Improving knowledge of how to provide effective hearing support, establishing caregiver responsibilities and increasing the resources for hearing support within LTCHs.

## IMPLICATIONS FOR REHABILITATION

- Interventions to aid family caregivers in providing hearing support for their relative with dementia should be designed to address gaps in capabilities, opportunities and motivations, as barriers are present in all three constructs.
- Audiologists should signpost family caregivers to educational resources about traditional hearing aids and assistive listening devices to be used within the long-term care homes (LTCH) and understand the benefits of domiciliary audiology appointments for LTCH residents.
- Care staff and family to discuss roles and responsibilities for hearing support and document these in care plans when the resident first moves to the LTCH e.g., accompanying resident to external audiology appointments.

## Background

The prevalence of dementia and hearing loss in long-term care home (LTCH) residents is high; approximately 74% [1] and 82% [2], respectively. Several studies indicate that hearing loss is highly prevalent in people with dementia (60–90%) [3,4], making it an important aspect of care for the vast majority of LTCH residents with dementia. Untreated hearing loss in residents with dementia can lead to communication difficulties [5], loneliness [6] and increased negative behaviours [7]. Supporting residents’ hearing can enhance their quality-of-life [8], but hearing loss often goes undetected [9], hearing aid use is low [10] and hearing support is typically not a priority in LTCHs [11].

Recent work recommends adopting a personalised approach to suit residents’ abilities and involving family with hearing support wherever possible [8,12,13]. Family caregivers (the term “family” is used to represent family caregivers, family members, or close friends who play a significant part in the residents’ life and care in the current study) may play a key role in the provision of hearing support within LTCHs. Family involvement can help to meet the emotional and physical needs of residents [14] and is a crucial aspect of “family-centred care”; a model acknowledging the important role that family plays in residents’ lives [15]. However, the family’s role within LTCHs remains ambiguous [16], and disagreements between family and LTCH staff can occur concerning caring responsibilities and what constitutes “good” care [17].

As most residents require caregiver support for hearing-related needs [10,18], consideration of the family’s role is essential. Little is known about the experiences of family in providing hearing support to LTCH residents with dementia. A small number of case studies suggest family involvement facilitates residents’ use of hearing devices, e.g., changing hearing aid batteries [19] and accompanying the resident to audiology appointments [7]. However, under 2% of LTCH staff believe family to be responsible for hearing support, and fewer than 14% believe it to be a collaborative responsibility [20]. It remains uncertain whether family perceive hearing support as their responsibility (e.g., are they motivated?), how they work – or do not work – alongside LTCH staff (e.g., do they have the opportunities?) and whether they have the knowledge and skills (e.g., are they capable?) to provide effective hearing support.

This study aims, for the first time, to explore the capabilities, opportunities and motivations of family caregivers in providing hearing support to LTCH residents with dementia. This study uses the Capabilities, Opportunities, and Motivations model of Behaviour change (COM-B) (a central component of the Behaviour Change Wheel) [21] to explore why family caregivers do, or do not, provide hearing support. We use this evidence to recommend interventions strategies that could address the barriers and improve hearing support within LTCHs.

### Research questions

- To what extent do family caregivers provide hearing support to their relative living with dementia in a LTCH?
- What hearing support methods do family caregivers use with residents with dementia and what are their views on these different methods?
- What are the capabilities, opportunities and motivations of family caregivers to provide hearing support for their relative living with dementia in a LTCH?
- Which interventions could be used to help family caregivers provide hearing support to their relative with dementia?

## Materials and methods

The study received approval from the University of Manchester Research Ethics Committee (2021-11502-19581). Data collection occurred between May 2021 and January 2022. Participants provided written informed consent through online forms and received £5 High-Street e-vouchers for the survey and an additional £15 High-Street e-voucher after the interview. Survey data were collected and managed using REDCap [22], and interviews were conducted remotely on Zoom by one researcher (HC).

### Study design

To address the limited research in this area, an exploratory two-stage approach was used: a cross-sectional survey (Stage 1) and semi-structured interviews (Stage 2). This combination of qualitative and quantitative methods allowed for a comprehensive, multi-perspective study.

The survey and interview questions were co-designed with *N* = 2 Public and Participant Involvement (PPI) advisors (Co-authors AC-T, SB), who collaborated on this study because they had lived experience of being family caregivers of LTCH residents with dementia and/or hearing loss. PPI entailed a one-hour Zoom session with PPI advisors and study researchers (HC, RM) to discuss the study aims and methods. PPI advisors also reviewed the drafted survey and interview questions in their own time so that they could edit or add to these materials. They were also involved in reading and editing the final manuscript. Amendments based on PPI feedback included adding open-ended questions to the survey and revising the wording of some items and questions.

### Theoretical framework

The study design was guided by the 6-domain COM-B Model [21]. This model suggests that to engage in behaviour, such as providing hearing support to residents with dementia, individuals need Physical and Psychological Capability, Social and Physical Opportunity, and Reflective and Automatic Motivation. To change behaviour, there is a need to modify capability, opportunity, and/or motivation accordingly. The 14-domain Theoretical Domains Framework (TDF) [23], which complements COM-B, was also employed for stage 2 interviews to delve into capabilities, opportunities and motivations in greater depth. Together, these models cover behavioural determinants, e.g., TDF’s “Knowledge” maps to COM-B’s “Psychological Capability.” This approach provides insight into why family caregivers do, or do not, provide hearing support to their relative, and also allows for intervention development by selecting relevant Intervention Functions from the Behaviour Change Wheel [21].

### Participants

UK-based family caregivers of LTCH residents with dementia and hearing loss, aged over 18, were recruited through convenience sampling due to COVID-19 lockdowns causing potential recruitment challenges. Survey recruitment involved sharing study posters and survey links through email to carer networks nationwide and posting on social media platforms. Out of the 96 attempted survey completions, 87 respondents met the inclusion criteria and completed the main body of the survey. On completion of the survey, respondents were asked to provide their name and email address should they wish to hear more about interviews. Twenty-seven participants expressed interest in a follow-up interview and were sent the interview information sheet. Six participants responded to the invitation and participated in the interviews.

### Procedure

#### Stage 1: Survey

Survey questions are available in Appendix A. Demographic information about family caregivers and their relative was collected first. “Hearing support” was defined broadly to include various methods used in LTCHs: “hearing aids or other hearing devices, using communication aids such as pictures or flashcards, or changing your communication techniques.” The survey incorporated a validated measure to gauge participants’ perceptions of capabilities, opportunities, and motivations related to hearing support [24] e.g., “I am physically able to provide hearing loss support for my relative/friend with dementia. For example: having the physical skills to insert hearing device, change batteries,” requiring participants to respond on an 11-point Strongly Disagree-Strongly Agree scale. Additional multiple-choice, checkbox and Likert-scale questions were included to explore capabilities, opportunities and motivations further: e.g., accessing audiology services and sharing caring responsibilities with care staff.

#### Stage 2: Interviews

For stage 2, one-to-one interviews were conducted, following the interview schedule outlined in Appendix B. The schedule was shaped by the COM-B Model and the TDF: “*Do you receive support from, or work collaboratively with, staff members?*” investigated COM-B’s “Social Opportunity” and TDF’s “Social cues,” for example. Interviews were audio-recorded, transcribed verbatim, proofread, and anonymized by HC.

### Data analysis

#### Stage 1: Survey

Quantitative data were analysed using IBM SPSS V.25 by HC. Descriptive statistical analysis (response percentages, means, and standard deviations) was conducted. A within-participants ANOVA was used to explore potential differences in participants’ six COM-B scores (Physical Capability, Psychological Capability, Reflective Motivation, Automatic Motivation, Physical Opportunity, Social Opportunity) and therefore identify key gaps in capabilities, opportunities, and motivations [24]. No significantly influencing outliers were observed in the boxplots and normal distributions were seen in the quantile-quantile plots. Sphericity assumption was violated (Mauchly’s Test, *p* = 0.005), so the Huynh-Feldt correction was applied. Due to missing responses in the physical opportunity items from 12 participants, the ANOVA was conducted with *N* = 75 participants.

#### Stage 2: Interviews

Lumivero NVivo V.12 [25] was used for qualitative analysis. Interviews were analyzed deductively and inductively. Direct summative content analysis [26], conducted by two authors (HC, RM) identified and categorized instances of the TDF domains in the transcripts. The two authors compared TDF coding counts, having substantial agreement (κ > 0.6) [27]. Prominent TDF domains (appearing in ≥60% of transcripts) were then mapped to their corresponding COM-B domains.

Themes were used to further specify the drivers of behaviour, relating to prominent TDF domains. Reflective thematic analysis [28] was conducted by three authors (HC, AC-T, SB). Thematic analysis is a qualitative method used to analyse recurring patterns or concepts within an interview dataset, relevant to the research questions. Themes are conceptualizations that are generated to represent the important underlying experiences, meanings or patterns within the data. The three authors conducted their analyses independently and compared their interpretations and themes during a discussion session. Potential themes and patterns were highly consistent, but due to the reflective nature of qualitative work, there were some discrepancies in the names of the themes. The authors discussed their theme names and reasonings before deciding on final themes in which all authors agreed to.

### Interventions

Based on the findings of the survey and interviews, we selected relevant Intervention Functions from the Behaviour Change Wheel that may address gaps in capabilities, opportunities and motivations [21]. Intervention functions and exemplar interventions are outlined in Table 3.

## Results

### Participant demographics

For both the survey and the interview stages, most participants were female (demographics are provided in Tables 1 and 2). The samples were predominantly White British. The mean age of survey participants was 37.4 years. Interview participants were notably older, with a mean age of 57.8 years. The ages of interview participants are provided alongside selected quotes, except for one participant who did not disclose their age. The relationship of the participants to a resident varied, with a significant proportion being daughters in the interview sample, and broader representation in the survey sample. Education levels varied, with a majority holding undergraduate degrees, or equivalent qualifications, for both the survey and interview stages.

**Table 1.** Family caregiver demographics.

Demographic	Survey (Stage 1) *N* (%) *N* = 87	Interview (Stage 2) *N* (%) *N* = 6
Gender		
Woman	52 (59.8%)	6 (100%)
Man	35 (40.2%)	
Ethnicity		
White British	45 (51.7%)	6 (100%)
Black/Black British	27 (31.1%)	
Asian/Asian British	13 (14.9%)	
Mixed/Multiple ethnic group	2 (2.3%)	
Age	*Mean* = 37.4 (*SD =* 12.2)	*Mean =* 57.8 (*SD* = 9.3)
20–40	63 (72.4%)	
41–60	11 (12.6%)	3 (50%)
61+	7 (8.1%)	2 (33.4%)
Did not answer	6 (6.9%)	1 (16.6%)
Relationship to resident		
Daughter/Son	28 (32.2%)	5 (83.3%)
Niece/Nephew	23 (26.4%)	
Granddaughter/Grandson	5 (5.8%)	
Sister/Brother	3 (3.4%)	
Husband/Wife/Partner	1 (1.2%)	
Cousin	4 (4.6%)	
Daughter-in-law	2 (2.3%)	1 (16.7%)
Unspecified e.g., “relative”	17 (19.5%)	
Did not answer	4 (4.6%)	
Frequency of visits to resident (prior to COVID-19 restrictions)		
Daily	3 (3.5%)	
Once a week or more	16 (18.4%)	2 (33.3%)
Once a month or more	31 (35.6%)	
Less than once a month	3 (3.5%)	
Unspecified e.g., “often” or “frequently”	17 (19.5%)	
Did not answer/ Relative not in care home prior to COVID-19 restrictions	17 (19.5%)	4 (66.7%)
Highest level of education		
No Qualifications	1 (1.2%)	
Diploma or equivalent	13 (14.9%)	2 (33.3%)
GCSE or equivalent	6 (6.9%)	1 (16.7%)
A-Level or equivalent	4 (4.6%)	1 (16.7%)
Undergraduate degree or equivalent	48 (55.2%)	2 (33.3%)
Postgraduate degree or equivalent	13 (14.9%)	
Prefer not to answer	2 (2.3%)	

*Note*: GCSE – academic qualifications taken in UK education, usually at 16 years old. A-Level – academic qualifications taken in UK education, usually at 18 years old.

**Table 2.** Resident demographics (reported by family caregivers).

Demographic	Survey (Stage 1) *N* (%) *N* = 87	Interview (Stage 2) *N* (%) *N* = 6
Dementia diagnosis		
Alzheimer’s disease	13 (14.9%)	2 (33.3%)
Vascular dementia	18 (20.7%)	
Mixed dementia	29 (33.3%)	3 (50.0%)
Dementia with Lewy bodies	12 (13.8%)	
Frontotemporal	6 (6.9%)	
Mild cognitive impairment	6 (6.9%)	
No formal diagnosis	1 (1.1%)	
Unknown	2 (2.3%)	1 (16.7%)
Stage of dementia		
Early	19 (21.8%)	
Middle	53 (60.9%)	5 (83.3%)
Late	13 (14.9%)	
Unknown	2 (2.3%)	1 (16.7%)
Hearing loss severity		
Mild	20 (23.0%)	
Moderate	47 (54.0%)	5 (83.3%)
Severe	19 (21.8%)	1 (16.7%)
Unknown	1 (1.1%)	
Level of care received		
Low-level	4 (4.6%)	1 (16.7%)
Mid-level	48 (55.2%)	5 (83.3%)
High-level	35 (40.2%)	
Time since moving into LTCH	*Mean months* = 31.3 (*SD =* 27.0)	*Mean months =* 19.2 (*SD =* 20.7)
Less than 1 year	17 (19.5%)	4 (66.7%)
1–2 years	18 (20.7%)	
2–3 years	14 (16.1%)	1 (16.7%)
4–5 years	6 (6.9%)	1 (16.7%)
Over 5 years	7 (8.1%)	
Unspecified e.g., “long time”	9 (10.3%)	
Did not answer	16 (18.4%)	
LTCH type		
Residential	35 (40.2%)	5 (83.3%)
Nursing	34 (39.1%)	
Dementia specialist	17 (19.5%)	1 (16.7%)
Did not answer	1 (1.1%)	
LTCH ownership		
Private company	48 (55.2%)	5 (83.3%)
Local authority	22 (25.3%)	
Charity/Voluntary	15 (17.2%)	1 (16.7%)
Don’t know	1 (1.1%)	
Did not answer	1 (1.1%)	

*Note*: Low-level care – resident generally independent. Mid-level care – resident requires assistance with care, independent with other activities. High-level care – resident needs full assistance, may receive care from a nurse. Residential LTCHs – accommodation, meals, personal care provided. Care Home with Nursing – registered nurses for residents with complex health needs employed. Dementia Specialist Homes – for residents with advanced cognitive and behavioural needs. Privately funded – LTCHs owned by private companies. Local Authority funded – LTCHs owned by UK district, borough or county council. Charity/voluntary owned – LTCHs run by charities, volunteers or not-for-profit companies.

### Stage 1: Survey

#### Providing hearing support (behaviour)

**Figure 1.**
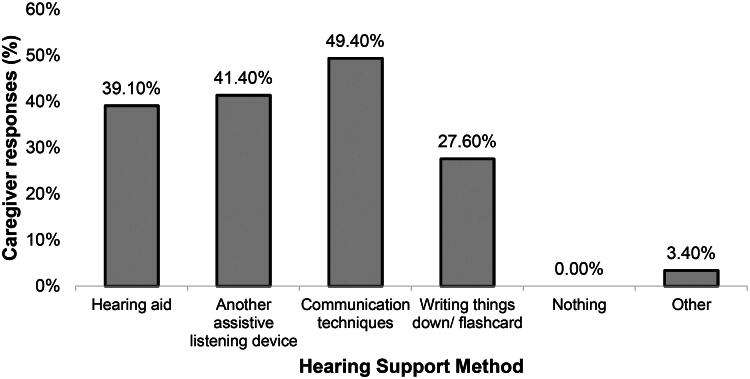
Family caregivers’ methods of providing hearing support to residents with dementia. *Note*. “Other” responses included: “flashing phone light.” Additional open-ended responses clarified “communication techniques” as: “eye contact and speaking louder,” “speak more clearly” and “hand gestures that mirror the word I’m trying to convey.”

Family caregivers scored an average of 6.0 (*SD* = 2.2) on a 0–10 scale (Strongly Disagree-Strongly Agree) regarding providing hearing support to their relative with dementia. When visiting their relative, participants reported providing hearing support “almost every time” (42.5%), followed by “every time” (36.8%), “over half the time’ (12.6%), “less than half the time” (5%), and “never” (4.6%). However, nearly 20% of participants did not report how often they visit their relative. Around half reported using communication techniques to support their relative (49.9%). Among residents with a hearing device (40.25%), 60.9% of family caregivers reported testing or checking the device. Figure 1 displays the methods of hearing support used by family caregivers. More than 40% (42.5%) of participants reported using a combination of methods, as they selected multiple responses.

#### Audiology services

Just over half (55.2%) of participants’ relatives had attended audiology appointments since moving into the LTCH and 67.8% had their hearing checked upon admission. Few (16.1%) provided a response to the frequency of these appointments, but they typically occurred annually or bi-annually, e.g., “2 x year before covid.” Audiology appointments took place in the community (48.7%) or in the LTCH (43.5%), 7.7% were unsure of the location. Five open-ended responses highlighted difficulties with maintaining residents’ hearing aids during COVID-19 restrictions. Either family (49.3%) or LTCH staff (48%) accompanied residents to appointments, only a small percentage (2.7%) of residents went alone. For the 60.9% of residents who had earwax removed, this was performed by an LTCH nurse (49.1%), General Practitioner (28.3%), or audiologist (18.9%).

#### Responsibilities for hearing support

Only 15.3% of participants saw themselves as responsible for supporting residents’ hearing, with care staff (35.2%) and nurses (35.2%) considered equally responsible. Collaborative responsibility was perceived by just 10.6%, and 1.2% believed residents to be responsible for their own hearing needs. “Other” (2.4%) included “no-one as far as I can tell.” In contrast, family viewed themselves as most responsible for arranging audiology appointments (52.9%), followed by LTCH staff (31.8%) and residents themselves (1.2%). Collaborative responsibility for audiology appointments was perceived by 14.1% of participants.

#### Knowledge development

About 63.2% wanted to know more about how they can support their relatives’ hearing.

#### Hearing aids

**Figure 2.**
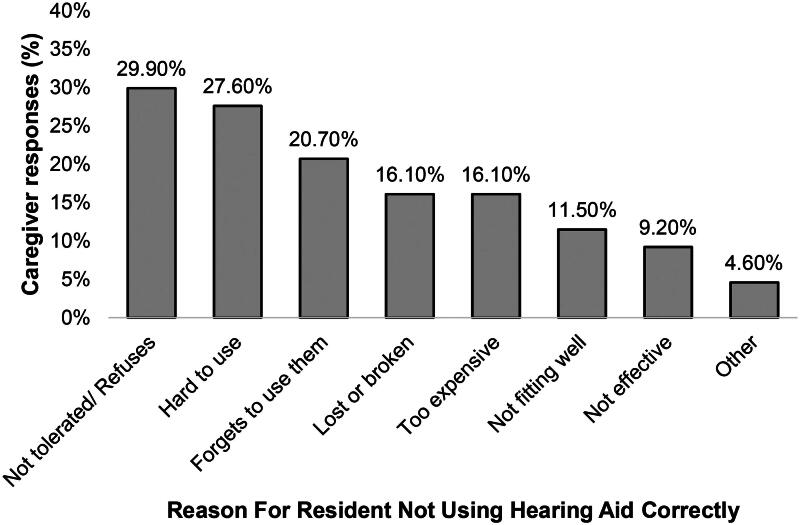
Reasons given for incorrect use of hearing aids by residents with dementia. *Note*: Open-ended “Other” responses (4.6%) included “*mum wouldn’t cope with placing a hearing aid*” and “*sometimes, even with hearing aids, it is difficult to communicate*.”

Participants scored near the mid-point for whether they believed their relative to be able to use a hearing aid correctly (*M =* 5.6, *SD =* 2.5). Explanations for residents’ incorrect use are provided in Figure 2, with 32.2% of participants reporting multiple difficulties. Participants scored *M* = 6.2 (*SD* = 2.2) on a 0–10 scale in response to whether they believe adaptations are needed to hearing support because of their relative’s dementia.

#### The LTCH context

Participants scored *M* = 6.2 (*SD =* 2.1) on a 0–10 scale as to whether they agreed that their relatives’ LTCH is sensory friendly. Comments included *“mostly carpeted”* and “*the nursing home is a little too quiet*.” Just over half (54%) said that a “Hearing Champion” staff member worked in their relative’s LTCH. Participants scored *M* = 6.2 (*SD =* 2.6) on a 0–10 scale as to whether they work alongside LTCH staff to provide hearing support.

#### COM-B domains

**Figure 3.**
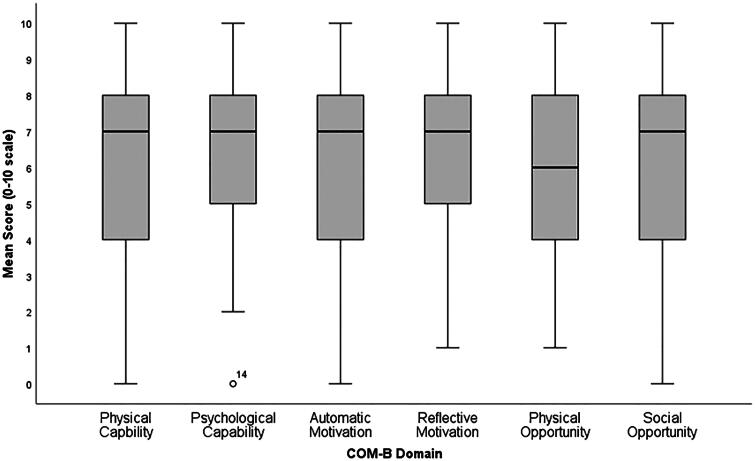
Box-and-Whisker plot displaying the distribution of the scores for the six COM-B domains. The Central black line shows the median score for each domain, the grey box encompasses the interquartile range (IQR) and the whiskers display the range of responses. There is one outlier for the domain Psychological Capability, which is not influential.

Scores for perceived COM (0–10) are reported in Table 3 and a box-and-whisker plot of these results is shown in Figure 3. A within participants ANOVA revealed no significant differences between domain scores (*F*(4.49, 318.89) = 1.85, *p* = 0.110).

### Stage 2: Interviews

Interviews lasted approximately 50 min. Interview participant (*N* = 6) demographics are shown in Tables 1 and 2.

One family caregiver’s relative frequently used a hearing aid. Two reported infrequent use of hearing aids and three reported non-use. One resident also used an assistive listening device alongside a hearing aid. Half of participants interviewed used communication techniques to support their relative’s hearing e.g., lipreading, and one wrote things down to aid communication.

*Knowledge*, *Environmental Context & Resources*, *Optimism* and *Social/Professional Role & Identity* were prominent Theoretical Domains, corresponding to *Psychological Capability*, *Physical Opportunity* and *Reflective Motivation*. Exploratory themes are outlined below in the context of each domain. All themes reflected the difficulties/barriers that family experience when providing hearing support to their relative. Potential interventions to address these barriers are explored in Table 3.

#### Knowledge (psychological capability): uncertainty about how to provide effective hearing support

Participants were unsure of the best approach to support their relatives’ hearing due to residents’ difficulties with traditional hearing aids (see *Optimism* below). Most were unaware of alternative methods. Although all wanted to know more about how best to support their relatives’ hearing, they were unsure of where they could access information specifically for supporting hearing loss in people with dementia:

I would like to know more. You know, it’s one of those things that I think she’s having to live with and I don’t really know how to help her. (FC-6, 71 yrs)

Other than hearing aids is there anything else, possibly? (FC-2)

There were also gaps in procedural knowledge of hearing aid management:

Is it red for right? Green for left? I don’t know. (FC-5, 61 yrs)

I must have put them [hearing aids] in the wrong ears, and they didn’t work. I don’t know but I really struggled with them. (FC-2)

#### Environmental context & resources (physical opportunity): poor collaborations between LTCHs and audiology services

Five participants discussed wide-ranging difficulties when organising audiology appointments for residents. Family caregivers reported that their relative was required to attend external clinics, rather than appointments within the LTCH. This presented complications in arranging transportation for residents with mobility problems, and residents’ distress when visiting unfamiliar settings:

I think it’s more the difficulty in arranging her to be referred and then getting an appointment […] there’s no way she’d get into my car now […] it’s quite obvious that her mobility isn’t very good at all. (FC-6, 71 yrs)

The lack of opportunity to access dementia-appropriate audiology services influenced family’s engagement on behalf of their relative:

We made the decision not to take her to any hearing tests because it would just be pointless […] She wouldn’t cope. She would definitely be stressed… She just wouldn’t know what was going on. (FC-2)

#### Environmental context & resources (physical Opportunity): low priority of hearing loss in LTCHs

Hearing loss was of a low priority compared to other physical and mental health conditions that residents live with:

She complains about things, and then she does say her ears are sore sometimes, or she can’t hear, then she goes onto something else… it’s difficult. (FC-5, 61 yrs)

…Having her ears syringed is a low priority thing. (FC-3, 53 yrs)

The prioritisation and impact of not addressing basic care needs (e.g., hydration and pressure care) was discussed. Because of this, participants believed hearing to be deprioritised within LTCHs by themselves, staff, and sometimes the residents themselves: Untreated and undertreated hearing loss does not have as immediate and serious consequences as suboptimal management of some other health conditions;

Food, yes, drink, stopping people from falling. You know, there’s all these things aren’t there? And so, you know, if you can get by…by shouting and using a pen and paper, then… it [hearing support] will go down the list. (FC-1, 58 yrs)

#### Environmental context and resources (physical opportunity): impact of COVID-19 restrictions

Five participants stressed the impact of COVID-19 restrictions within LTCHs on their ability to communicate effectively with their relative and support their hearing needs. Face mask requirements meant that residents could not lip-read or recognise facial cues. This had drastic effects for residents who rely solely on these methods, impacting relationships and increasing agitation:

Conversations are just so difficult, bordering on impossible now [due to face masks]. (FC-2)

The mask thing is just so bad… I try, you know, outside and I say to her, I repeat it. And if she still doesn’t get it, I pull the mask down and just say. And she gets it because she can see my lips moving and she can hear better. (FC-6, 71 yrs)

Window and/or pod visits were physical barriers that impaired communication between residents and family. Again, use of communication techniques was hindered:

Trying to listen through this [Amazon] Echo-ey thing… well I gave up in the end… it was just too upsetting to be honest to just go through that for half an hour when you spent a good 20 minutes of that just repeating yourself and trying to get, trying to get some sense of conversation. (FC-6, 71 yrs)

Similar experiences were reported during attempts to video call with residents:

It’s upsetting for us and it’s confusing for her [resident] and okay, she may forget afterwards, but that feeling… I tend to think the feelings of confusion or anger can probably stay with them, but they won’t know why … they’ll just sort of maybe feel uncomfortable. (FC-5, 61 yrs)

Moreover, existing audiology appointments for hearing tests, check-ups and hearing aid maintenance were disrupted due to additional precautions in place for LTCHs:

If they won’t have the audiologist or whatever in the care home at the minute, it just gets too difficult trying to get an appointment. Trying to get her out… when she’s having to wear masks and all of this… she’s confused before she gets there and upset. (FC-5, 61 yrs)

#### Social/professional role & identity (reflective motivation): lack of clearly defined responsibilities for hearing support

Perceived responsibilities for hearing support varied between family and staff caregivers, depending on the specific task. Family typically believed themselves to be responsible for taking residents to audiology appointments:

I’ve always taken over really. So, you know, I’ve gone in and even though I work full-time, before COVID… it was me that took her [to audiology appointments]. If I was really busy, then they [staff] would do it. But you know, I wanted to take her, in a way. (FC-2)

Alternatively, managing residents’ hearing aids seemed to be the responsibility of staff. This was because family typically lacked procedural knowledge of hearing aids (discussed in *Physical Capability*) and were understandably not always present in the LTCH:

I felt bad because I’d have to go ‘[staff]! Can you come and put her hearing aids in please?’ [laughs] because I couldn’t do it. (FC-2)

I can’t be in her life 24/7. It’s just physically impossible. (FC-4, 46 yrs)

However, responsibilities for hearing loss support were not always well-defined meaning that taking ownership was sometimes difficult:

It’s hard to know, you know, who should be making… if she’s going to get another NHS-issued digital hearing aid, then who should contact audiology? You know? Should that now be somebody from the council or somebody from the care home? We’re not sure. (FC-1, 58 yrs)

They [staff] need to be putting the [ear]drops in. Well, we can’t do that. So, you’re reliant on the home and I think they said ‘we’ll do drops five days before you go’ [to have ears syringed]. Well really, I think she [resident] should have them in every day, but they’re not going to do that. (FC-5, 61 yrs)

#### Optimism (reflective motivation): difficulties that residents with dementia experience with hearing aids

All participants discussed difficulties with hearing aids for residents with dementia. Residents often misplaced their hearing aids and locating these could take several days. Hearing aids sometimes had to be replaced by family as they were lost and never found:

The other issue she had when the dementia started was that she kept losing them. She’d hide them. Even when she’d moved into the care home… Every week we’d spend a big portion of our time trying to find her hearing aids […] it just got so ridiculous. (FC-2)

Residents’ rejection of hearing aids became a prominent difficulty with dementia progression. Residents were unaware, or unaccepting, of their hearing loss, and experienced physical discomfort with having hearing aids in their ears:

We had a year of her [resident] not wearing the hearing aids, because she didn’t have a problem, so she says, so she would hide them. (FC-4, 46 yrs)

She just yanks them [hearing aids] out… the whole thing would fly across the room. But that never used to happen, that wasn’t early on, that was as her dementia progressed. (FC-2)

Although participants reported encouraging their relative to use hearing aids, motivation and optimism decreased with time:

She did [have hearing aids]. She doesn’t now in fact, we’ve sent them back to the hospital now because she’s… she wouldn’t manage it now. So, she has in the past, but it was too confusing for her. (FC-5, 61 yrs)

**Table 3.** Summary of key findings: survey COM-B domain scores, additional survey findings, prominent TDF domains, themes, intervention functions, exemplar interventions.

COM-B domain	COM-B score M (*SD*)	Additional survey findings	Prominent TDF domain	Theme (generated from qualitative data)	Intervention functions	Example intervention for family caregivers
Physical capability	6.5 (*2.4*)		N/A			
Psychological capability	6.6 (*2.4*)	63.2% want to know more about providing hearing support.	Knowledge	Uncertainty about how to provide effective hearing support	Education	Providing educational resources (booklets, leaflets) on hearing aid management (cleaning, inserting, checking etc.) for family to view within the LTCH. Provide information about alternative amplification devices.
Physical opportunity	5.9 (*2.2*)	55.2% of residents see an audiologist, 43.5% of appointments take place in the LTCH. 60.9% of residents have earwax removed, mostly by a nurse in the LTCH (49.1%).	Environmental context & resources	Poor collaborations between LTCHs and audiology services	Environmental restructuring Enablement	Family to accompany resident to audiology appointments for support. Encourage family to inform audiologist of residents’ preferences, any difficulties and need for flexibility (e.g., shorten test duration, family to accompany resident into testing booth).
				Low priority of hearing loss in LTCHs	Education	Increasing family’s awareness of the implications of unsupported hearing loss (anxiety, depression, agitation, falls, etc.) to boost its priority.
		Open-ended responses emphasized impact of COVID-19 lockdown on audiological care and impact of face masks.		Impact of COVID-19 restrictions	Environmental restructuring	Provision of transparent face masks for family to wear when at the LTCH to ease communication with residents e.g., allow for lip-reading and visual cues.
		Sensory friendliness of the LTCH: *M =* 6.2 (*SD =* 2.1), on a 0–10 scale)				
Social opportunity	6.1 (*2.7*)	Family working alongside staff to provide hearing support to residents with dementia: *M =* 6.2, (*SD =* 2.6), on a 0–10 scale. Variable open-ended responses on collaborating with LTCH staff.	N/A		To be boosted *via* addressing “social/ professional role and identity” (see below).	
Reflective motivation	6.7 (*2.1*)	Few family caregivers see themselves as responsible for hearing support in the LTCH (15.3%). Most see themselves as responsible for arranging audiology appointments for their relative (52.9%). Few believe hearing support provision (10.6%) or arranging audiology appointments (14.1%) to be a collaborative responsibility between themselves and staff.	Social/professional role & identity	Lack of clearly defined responsibilities for hearing support	Education, Persuasion	Staff and family to determine roles and responsibilities for hearing support and document these in care plans when the resident first moves to the LTCH.
		Residents with dementias’ correct use of hearing aids: *M* = 5.6 (SD = 2.5), on a 0–10 scale, due to refusal (29.9%), difficultly using (27.6%) or forgetting to use (20.7%).	Optimism	Difficulties that residents with dementia experience with hearing aids	Environmental restructuring, Education	Provision of low-cost, easy-to-use hearing devices (e.g., assistive listening/personal sound amplification devices) within LTCHs for residents to use in place of hearing aids, or in conjunction with hearing aids, if needed. Raising awareness of different types of hearing devices and ensuring that family know that they can be used and can be effective. Educating family on implementing a flexible, transitionary period for hearing device (hearing aid or other) uptake for residents with dementia who struggle with devices.
Automatic motivation	6.2 (*2.7*)		N/A			

## Discussion

This two-stage study explored the capabilities, opportunities and motivations [21] of family caregivers when providing hearing support to LTCH residents with dementia. Although the brief COM-B measure did not identify a particular domain to target, further synthesis of qualitative and qualitative results indicates that boosting psychological capability, physical opportunity, social opportunity and reflective motivation have the potential to bring about behaviour change in family caregivers that will improve hearing support for residents with dementia.

### Behaviour

Only half of family caregivers used communication techniques with their relative with dementia and hearing loss, despite this being the most reported method of hearing support. Adapting communication (speaking clearly, facing the person, etc.) is recommended for communicating with people with advanced dementia, irrespective of hearing status [13,29]. The inability to understand what is said is detrimental to the quality-of-life of residents with dementia and hearing loss [30], and communication techniques should therefore be used by family and staff to lessen the impact [13]. Proxy-reported use of hearing aids and assistive listening devices was around 40%, which is higher than evidenced in a systematic review (16.7%-33%) [8]. The current measure is likely an overestimation, as respondents did not have the option to state frequency or consistency of use. Alternatively, they may be unaware that their relative is not wearing hearing aids when they are not visiting. Hearing device “use” classification is debatable [31], and interviews highlighted that ownership does not equate to consistent, effective use.

Few participants supported their relative’s hearing with two or more methods. Multi-component sensory support is recommended for people with dementia [13], as addressing hearing *via* amplification alone is unlikely to be effective or well-received [32]. Appropriate support may include the use of hearing aids by residents alongside the use of communication techniques by staff and addressing the LTCH environment to facilitate both hearing and comprehension [13]. Increasing family caregiver’s provision of multi-component, person-centred hearing support is necessary for caring for people with advanced cognitive impairment and difficulties adapting to traditional hearing aids.

### Capability

Previous research argues for the importance of increasing LTCH staff knowledge (psychological capability) about hearing aids [11,33], however, the current study is the first to identify major gaps in family caregivers’ capabilities too. With both formal and informal caregiver groups lacking knowledge in this area, residents with dementia who depend entirely on caregivers for hearing needs may be left without support for their hearing. Family caregivers were also unaware of alternative methods, particularly when hearing aids were rejected by residents, consistent with Bott et al. [34] Caregivers must know that alternative methods exist for residents to benefit from these alternatives. Free and easily accessible resources that address many of the uncertainties around the provision of hearing support are available [35]. Supplying printed resources within LTCHs (*Education*) and raising awareness of existing hearing aid training materials (*Training*) [11] within LTCHs would be a low-cost, low-effort intervention for both family and staff who are motivated to know more.

### Opportunity

Current audiology services were deemed inaccessible for residents with dementia. Family described difficulties with wheelchair-accessible transportation and hearing tests being stressful for residents with advanced dementia, consistent with similar accounts from LTCH staff [18,36]. It is vital that residents have opportunities to access home-based audiology services, alongside community-dwelling people with dementia. Recommendations for adapting services for people with dementia e.g., reducing test durations, using meaningful sound stimuli, or conducting assessments at the person’s home [12] should be followed for LTCH residents (*Enablement* and *Environmental Restructuring*). Future investigations into whether such adaptations reduce the anxiety of LTCH residents and their family caregivers, thus increasing engagement in these services, whilst ensuring the reliability of the assessment, is needed. Research involving both audiologists and LTCH stakeholders on how best to develop these protocols would be ideal.

The low prioritisation of hearing and communication within LTCHs has been reviewed previously [11,34], however, this is the first instance of family caregivers providing insight as to why hearing support is deprioritised. As most residents with dementia have multimorbidity [37], competing care needs (physical comfort, falls, hydration, etc.) can take priority for family, staff and residents [38]. Increasing awareness of the implications of unsupported hearing loss in residents (depression, falls, agitation, etc.) through education for family caregivers may alter care priorities (*Environmental Restructuring via Education*).

Hearing loss was increasingly deprioritised due to COVID-19-related procedures. The results build on previous research on the impact of COVID-19 restrictions on connections and communication between LTCH residents with dementia and family [39], by incorporating the added difficulty of unsupported hearing loss. Current findings also support those of De Andrade & Landman [40] where they noted the impact of lockdowns on audiological support for LTCH residents. Face masks are still recommended for certain circumstances within LTCHs in the UK and will continue to impact on family caregivers’ ability to use communication techniques as a main form of hearing support. Transparent face masks improve speech intelligibility *via* visual input [41] and, when risk-assessed, are recommended for communicating with LTCH residents with dementia and/or hearing loss [42] (*Environmental Restructuring* and *Guidelines*). Whether caregivers use transparent facemasks is unknown. Further research on their use, acceptability and effectiveness for communicating with people with dementia and hearing loss would be beneficial.

### Motivation

Generally, family took responsibility for arranging and accompanying residents to audiology appointments – consistent with “arranging for outside experts” being the responsibility of family [43] – to mediate the stresses residents experience when attending external healthcare appointments. This may be due to LTCHs lacking the required number of staff to accompany residents on the 1:1 basis necessary for residents with mobility difficulties and/or behavioural symptoms [36]. Family typically believed LTCHs to be responsible for hearing aid maintenance, consistent with “technical” day-to-day care being a role that family believed to be the responsibility of staff in previous work [44]. This may be due to the gaps in procedural *knowledge* of hearing aid management reported by family caregivers. However, there were clear instances where responsibilities were unclear. Family caregivers and LTCH staff are caregiving groups with overlapping roles, which can lead to uncertainty when co-ordinating care, if planned poorly. Establishing role responsibilities, and documenting these clearly within care plans, so that all stakeholders are aware (*Education*) early in the resident’s move to a LTCH is necessary and may lead to regular audiology appointments, maintenance and subsequent use of hearing aids. Ultimately, we believe that the responsibility for providing care lies with the LTCH home, and that family members should not automatically be assumed to be responsible. However, for family carers who wish to remain part of the resident’s care team – as did many of the participants in this study – these discussions would be beneficial at the point when the resident is admitted into long-term care. Each person’s network of caregivers will be unique and family that prefer to be actively involved will therefore require the capabilities and opportunities to do so.

In line with previous research, LTCH residents with dementia struggled with traditional hearing aids [7,33,45] making it difficult for family caregivers to provide support with this aspect of hearing support. Residents’ misplacement, removal and rejection of hearing aids reduced family caregiver’s motivation to encourage and support hearing aid use. Provision of hearing devices that are low-cost and easy-to-use and manage e.g., assistive listening/personal sound amplification devices, within LTCHs may address some of the issues (*Environmental Restructuring*), thus increasing family’s motivation to provide support with amplification. Assistive listening devices have been recommended for use in LTCHs for residents who struggle to adapt to hearing aids [13,46], though only 16% of LTCHs have assistive devices available for residents [47]. The lack of awareness of alternative hearing devices may be a contributing factor to low use, therefore raising caregivers’ knowledge of amplification alternatives is vital (*Education*).

### Strengths and limitations

The current study is the first ever to focus on the capabilities, opportunities and motivation of family caregivers when providing hearing support to LTCH residents with dementia. The triangulation of exploratory results from both quantitative and qualitative approaches provides insight and lays the groundwork for future research. Although the data were collected during the COVID-19 lockdown period, we argue that the results are still relevant today. For example, residents with dementia continue to have complex health needs that are likely to be deprioritised compared to hearing loss, home-based audiology assessments are still not routine and many people with dementia continue to struggle with traditional hearing aids. At the time of publication of this study, face masks were not mandatory in UK care homes. However, face masks are likely to be reintroduced during future epidemics and pandemics to protect staff, residents and visitors against infections. It may be beneficial to repeat this study now that lockdown is over. This would likely result in facemasks being less of an issue because they are not worn as often. However, we would expect the remaining results to be replicated.

We have previously studied the capabilities, opportunities and motivations of care staff in providing hearing support [20,36]. However, we are yet to include the views of residents which is a limitation. As researchers were not permitted to enter LTCHs at the time of COVID-19 restrictions, face-to-face interviews with residents were not possible. Furthermore, barriers highlighted in this study regarding videocalls with people with dementia and hearing loss precluded our ability to involve them in remote interviews. Logically, the next stages would involve a similar study with residents themselves.

A further limitation is the small sample size who took part in the interviews. A larger number of interview participants may have resulted in additional themes or prominent domains. In addition, the small sample size for the survey and interview stages may have had an impact on the generalisability of the findings, especially as all interview participants were White British females and the study sample is therefore not representative of all family caregivers. Again, data collection during COVID-19 limited the recruitment of LTCH stakeholders. In future, LTCH research should be mindful to involve family caregivers, as well as care staff, to develop our understanding of their role in providing hearing support to residents. It may also be beneficial to conduct a similar study with family caregivers of people with other long-term health conditions, who would benefit from hearing support.

## Conclusions

Qualitative exploration highlights difficulties in psychological capability (knowledge), reflective motivation (optimism) and physical opportunity (environmental context & resources). Quantitative investigation also supports the need for improvement in these domains. However, further study would be beneficial to investigate family’s physical capability, social opportunity and automatic motivation, specifically. Providing hearing support to residents with dementia can be challenging. Interventions involving family caregivers if they wish, should be multi-component, ideally through education about hearing devices and implications of untreated hearing loss, providing clear face masks when necessary and early establishment of caregiver responsibilities.

## Supplementary Material

Supplemental Material
