# Supplementary material for: “I don’t really know how to help her.” Family caregivers’ capabilities, opportunities and motivations to provide hearing support to long-term care home residents with dementia
Source: Disabil Rehabil. 2024 Aug 7;47(7):1817–27. doi: 10.1080/09638288.2024.2384630 (PMC11974921; doi:10.1080/09638288.2024.2384630)
Supplement: Supplemental Material [file IDRE_A_2384630_SM8460.docx]

**Appendix A: Copy of survey.**

DEMOGRAPHICS:

Gender:

• Woman

• Man

• Prefer to self-define as: [COMMENT BOX]

• Prefer not to answer

Age:

• [COMMENT BOX]

• Prefer not to answer

Ethnicity:

• White

• Mixed/ multiple ethnic group

• Asian/ Asian British

• Back/ African/ Caribbean/ Black British

• Other ethnic group [COMMENT BOX]

• Prefer not to answer

Level of education (select your highest)

• Postgraduate qualification (Masters or Doctorate)

• Undergraduate degree or equivalent

• Diploma certificate or equivalent

• A-Level or equivalent

• GCSE or equivalent

• No Qualifications

• Other [COMMENT BOX]

• Prefer not to answer

Your relationship to care home resident: [COMMENT BOX]

Prior to COVID-19 restrictions, how often did you typically visit your relative/ friend living in a care home: [COMMENT BOX]

THE CARE HOME:

Your relative/ friend’s care home is owned by a:

• Local authority

• Private company

• Charity/ Voluntary

• Don’t know

Type of home they live in:

• Residential Care Home

• Care Home with Nursing

• Dementia Specialist Home

• Don’t know

In your opinion, is their care home a sensory friendly environment effective for residents with hearing loss and dementia (e.g., quiet enough, allows for hearing impaired residents to communicate well, not overstimulating):

Strongly Disagree 0 1 2 3 4 5 6 7 8 9 10 Strongly Agree

Any other comments on the care home environment suitability for people with dementia and hearing loss? [COMMENT BOX]

Is there is a ‘hearing loss champion’ staff member in your relative/ friend’s care home:

• Yes

• No

• Don’t know

RESIDENT INFORMATION:

What is your relative/friend’s dementia diagnosis (if known)?

• Alzheimer’s Disease

• Vascular Dementia

• Mixed Dementia

• Dementia with Lewy Bodies

• Frontotemporal Dementia

• Mild Cognitive Impairment

• Not formally diagnosed

• Unknown

• Other: [COMMENT BOX]

What is their stage of dementia (if known)?

• Early

• Middle

• Late

• Unknown

What is your relative/ friend’s hearing loss severity (if known)?

• Mild

• Moderate

• Severe

• Unknown

Any other comments that you would like to add on your relative/ friend’s diagnoses? [COMMENT BOX]

How long has your relative/friend lived in a care home for: [COMMENT BOX]

What was the reason for them moving into the care home: [COMMENT BOX]

What level of care does your relative/friend currently receive (if known):

• Low-level support (for example, they are generally independent with personal care needs)

• Mid-level support (for example, they require some assistance with care needs but independent with other activities)

• High-level support (for example, they require full assistance with all care needs and may receive care from a registered nurse)

• Don't know

HEARING LOSS SUPPORT FOR YOUR RELATIVE/ FRIEND LIVING WITH DEMENTIA:

“The following questions are about hearing loss support for your relative/ friend. Hearing loss support includes: hearing aids or other hearing devices, using communication aids such as pictures or flashcards or changing your communication techniques to help”

Who is responsible for providing hearing loss support for your relative/ friend?

(For example, changing the hearing device batteries, providing them with flashcards etc.)

• Care Staff

• Nurses

• Relatives/ Friends

• Resident

• Combined Effort/ Collaborative

• Other: [COMMENT BOX]

I provide hearing loss support to my relative/ friend living with dementia:

Strongly Disagree 0 1 2 3 4 5 6 7 8 9 10 Strongly Agree

What do you use to support your relative/ friend with their hearing loss? Select all that apply

• They wear a hearing aid

• They wear another assistive listening device

• I write things down / use flashcards with them

• I use communication techniques (e.g., speaking louder, speaking slower, standing face-to-face with your relative/ friend) with them

• Nothing

• Other: [COMMENT BOX]

If you alter your communication strategies so that your relative/ friend with hearing loss and dementia can better understand you, please explain how/ provide examples: [COMMENT BOX]

When you visit, how often do you provide hearing loss support for your relative/ friend with dementia:

• Every time I visit

• Almost every time I visit

• Over half of the times I visit

• Less than half of the time

• Never

My relative/ friend with dementia can use a hearing aid or other hearing assistive device correctly:

Strongly Disagree 0 1 2 3 4 5 6 7 8 9 10 Strongly Agree

If not, why?

• Not Fitting Well

• Hard to Use

• Not Tolerated/ Refuses

• Too Expensive

• Lost or Broken

• Not Effective

• Resident Forgets to Use Them

• Other: [COMMENT BOX]

Hearing loss support needs to be adapted for my relative/ friend because of their dementia or cognitive impairment:

Strongly Disagree 0 1 2 3 4 5 6 7 8 9 10 Strongly Agree

If so, please explain why: [COMMENT BOX]

Do you carry out testing or checking of your relative/ friend’s hearing aid or hearing device when you visit?

• Yes

• No

• My relative/friend does not use a hearing device

I am physically able to provide hearing loss support for my relative/ friend with dementia

(For example: having the physical skills to insert hearing device, change batteries)

Strongly Disagree 0 1 2 3 4 5 6 7 8 9 10 Strongly Agree

I am psychologically able to provide hearing loss support for my relative/ friend with dementia (For example: remembering to check that a hearing device is working, understanding the impact of untreated hearing loss for your relative/ friend)

Strongly Disagree 0 1 2 3 4 5 6 7 8 9 10 Strongly Agree

I would like to know more about how I can support my relative/ friend with their hearing loss:

• Yes

• No

Any other comments on this: [COMMENT BOX]

Providing hearing loss support for my relative/ friend with dementia is something that I do automatically (For example: You don’t think about it before doing it, you want to do it)

Strongly Disagree 0 1 2 3 4 5 6 7 8 9 10 Strongly Agree

I am motivated to provide hearing loss support to my relative/ friend with dementia (For example: you make plans to provide this support because you think it is needed)

Strongly Disagree 0 1 2 3 4 5 6 7 8 9 10 Strongly Agree

I have the physical opportunity to provide hearing loss support for my relative/ friend with dementia (For example: having enough time, having devices and aids provided in the care home)

Strongly Disagree 0 1 2 3 4 5 6 7 8 9 10 Strongly Agree

I have the social opportunity to provide hearing loss support for my relative/ friend with dementia (For example: working together with care staff, support from others)

Strongly Disagree 0 1 2 3 4 5 6 7 8 9 10 Strongly Agree

I work alongside care staff to provide hearing loss support to my relative/ friend with dementia:

Strongly Disagree 0 1 2 3 4 5 6 7 8 9 10 Strongly Agree

Do you have any further comments on your experience of co-operating with care staff to provide this support? [COMMENT BOX]

ACCESS TO EXTERNAL SERVICES:

Did your relative/ friend have their hearing checked when they moved into the home?

• Yes

• No

• Don’t know

Does your relative/ friend have appointments with external audiology services since moving into the care home?

• Yes

• No

• Don’t know

Who is responsible for arranging audiology visits for your relative/ friend?

• Care home staff

• Me or another family member/ friend

• Combined effort/ collaborative

• My relative/ friend themselves

• Other (please state): [COMMENT BOX]

Any other comments on the arrangement of audiology appointments?

[COMMENT BOX]

Generally, how often does your relative/ friend see an audiologist? (e.g., every 12 months.)

• [COMMENT BOX]

• They do not see an audiologist

• Don’t know

Appointments with the audiologist take place:

• In the care home – the audiologist visits my relative/ friend

• At an audiologist in the community

• They do not have appointments

• Don’t know

If your relative/ friend visits an external audiologist in the community, who accompanies them to these appointments?

• Care home staff

• Me or another family member/ friend

• They go by themselves

• Other (please state): [COMMENT BOX]

Any other comments on your relative/ friend’s appointments with audiology services: [COMMENT BOX]

Does your relative/ friend have their earwax removed?

• Yes

• No

• Don’t know

If yes, who performs the earwax removal?

• Audiologist

• GP

• Care home nurse

• Other (please state): [COMMENT BOX]

• Don’t know

**Appendix B: Interview schedule**

Introduction:

“This project is concerned with the way in which hearing loss support is provided for care home residents living with dementia. This includes using hearing aids or other devices, using communication aids like flashcards or any other method that helps. We are interested in hearing your views as a family members/close friend of a resident affected by both hearing loss and dementia and how you might support them when you visit.

The discussion will last for around 45 minutes, and you are free to leave at any time. You do not have to answer anything you don’t want to.

Do you have any questions for me at this point?

You’ve already completed the consent form. To check again, are you happy to continue with this discussion?

And are you happy for this to be audio recorded?”

| Please can you introduce yourself and tell me a bit about your relative/friend [*delete as appropriate*] who lives in the care home?  Prompts: information regarding relative/ friend’s health and need of support? How long have they lived in a care home for? Do they have a diagnosis of dementia or cognitive impairment etc? How severe is their hearing loss?  Can you give me a bit of information on how hearing loss support is provided for your relative/ friend in the care home? What is used or done to help with their hearing? | |
| --- | --- |
| **COM-B domain** | **Theoretical Domain (prompts)** |
| **Reflective motivation**  ‘What do you think about the quality of hearing loss support in care homes for your relative/friend with dementia?  Do you think that it is done well? Why/why not?’  ‘What are the benefits of providing hearing loss support?  To relative/friend with dementia?  To you?’  ‘What do you think are the drawbacks, if any, for not providing hearing loss support to your relative/friend with dementia?’  ‘Do you see hearing loss support for your relative/friend as something that you are personally responsible for?  If not, who is and why?’ | Optimism  Beliefs about consequences  Intentions  Goals  Social/ professional role and identity  Beliefs about capabilities |
| **Automatic Motivation**  ‘What helps, or would help, in developing a routine to ensure that hearing loss support is always provided for your relative/friend?  To help you work alongside care staff?’  ‘Do you find any aspects of providing this hearing loss support stressful or difficult?’ | Reinforcement  Emotion |
| **Physical capability**  ‘To what extent do you have the physical ability to provide hearing loss support?  For example, the *physical skills* to change hearing aid batteries or use loop systems within the care home?’ | Skills |
| **Psychological capability**  ‘Would you say that you have knowledge surrounding hearing loss and dementia? And how does that impact on your ability to provide the hearing support?  Are you aware of the different modes of hearing support available for your relative/friend?’  ‘Would you like to know more about hearing loss and how to provide hearing loss support for your relative/friend?’ | Knowledge  Memory, attention, decision process  Behaviour regulation |
| **Physical opportunity**  ‘To what extent does the care home provide you with opportunities to provide hearing loss support for your relative/friend with dementia?  Enough time when you visit, enough resources within the home etc.’  ‘Can you tell me a bit about your relative/friend’s care home’s environment? Does it allow you to communicate well with your relative/friend when you visit? E.g., Is it loud/ quiet? In communal areas/ bedroom?’ | Environmental context and resources |
| **Social opportunity**  ‘Do you receive support from or work collaboratively with staff members in the home to provide hearing loss support for your relative/friend?’  ‘How do arrangements with external services, such as GPs or audiologists work regarding hearing loss support?  Prior to COVID restrictions?’  ‘Any changes since they have moved into the care home? Easier/ harder to access now than before when they lived in the community?’ | Social cues/ influences |
| **Open questions:** | |
| In your opinion, what do you think is the best way to provide hearing loss support for your relative/friend?  Is there anything you would like to add to this discussion? | |
